# Unveiling the role of miRNA in laryngeal squamous cell carcinoma progression: a retrospective study

**DOI:** 10.1007/s00405-025-09306-y

**Published:** 2025-03-28

**Authors:** Nejmiye Akkuş, Elif Kaya Çelik, Suleyman Ozdemir, Emrah Sapmaz

**Affiliations:** 1 Faculty of Medicine, Department of Medical Genetics, Tokat Gaziosmanpasa University, Tokat, Turkey; 2 Faculty of Medicine, Department of Otolaryngology Head and Neck Surgery, Tokat Gaziosmanpasa University, Tokat, Turkey; 3 Faculty of Medicine, Department of Pathology, Tokat Gaziosmanpasa University, Tokat, Turkey

**Keywords:** LSCC, miRNA-21, miRNA-802, miRNA-29, miRNA-9

## Abstract

**Objective:**

MicroRNAs (miRNAs) have emerged as promising biomarkers for diagnosis and prognosis in laryngeal squamous cell carcinoma (LSCC). This study investigates the expression patterns of specific cancer-associated miRNAs in early and advanced stage LSCC to evaluate their potential role in disease progression.

**Methods:**

The expression levels of miRNA-21, miRNA-802, miRNA-29 and miRNA-9 were analysed in formalin-fixed paraffin-embedded (FFPE) tissues from 44 LSCC patients (20 early stage, 24 advanced stage) using quantitative multiplex RT-PCR. Tissue samples were analysed retrospectively.

**Results:**

The expression level of hsa-miR-9a expression was significantly upregulated (5.2-fold) in advanced stage patients compared to the early stage group (*p* = 0.0088). Similarly, hsa-miR-802a showed significantly higher expression (9.2-fold) in advanced stage patients compared to early stage patients (*p* = 0.005). No significant differences in miRNA-21 and miRNA-29 expression levels were observed between the groups.

**Conclusion:**

As a result of the study, elevated miRNA levels in advanced stage patients can be used in targeted therapy and evaluated as a treatment option in LSCC patients.

## Introduction

Laryngeal cancer accounts for one-third of all head and neck squamous cell carcinoma (HNSCC) and is the sixth most common type of cancer in the World [1]. Laryngeal squamous cell carcinoma (LSCC), remains a significant disease burden worldwide, accounting for approximately 160,000 new cases annually and 2.5% of all tumors in men [2]. With the development of diagnostic tools in laryngeal cancer, early diagnosis and the preservation of laryngeal functionality have been the main goals. However, reviewing the literature reveals that treatment responses vary widely among patients [3]. Recently, studies have investigated the detection of biomarkers in laryngeal tumor tissue that may help to better characterize LSCC and identify the patient groups that may benefit from specific treatment approaches such as radiotherapy (RT), chemoradiotherapy (CRT), or immunotherapy [4, 5]. The critical role of miRNAs in tumorigenesis and their potential use as prognostic/diagnostic biomarkers have made these molecules an important research topic [6].

MicroRNAs (miRNAs), short non-coding RNA molecules, consist of approximately 22 nucleotides [7]. After binding to target messenger RNA(mRNA) molecules, eukaryotic miRNAs affect the protein translation step by causing mRNA degradation, stopping translation, or regulating mRNA translation [8, 9]. Since one miRNA can target approximately 400 different mRNAs, miRNAs are thought to be an important regulatory mechanism through their direct effects on transcription and translation [10]. A pathology that develops in miRNAs can cause many diseases such as cancer, metabolic disorders, inflammatory, cardiovascular, neuro-developmental, and autoimmune diseases [11].

The fact that miRNAs are readily detectable biomarkers in cancer diagnosis and prognosis has been demonstrated by the isolation of miRNAs from patients’ peripheral blood [12, 13]. Considering the challenges of standard tissue biopsies, sampling from biofluids offers a less invasive alternative for patients [14]. miRNA analysis in biological fluids offers advantages over formalin-fixed paraffin-embedded (FFPE) material, including lower cost and faster results [15, 16].

In the literature, the microRNA-802 (miRNA-802) gene located on chromosome 21 has been shown to be effective in the prognosis of laryngeal cancer. miRNA-802, a tumor suppressor gene, has an inhibitory effect on the formation and development of tumors. Previous studies have shown that miRNA-802 levels are reduced in some cancer tissues, including LSCC [17]. Another molecule that has been identified to play a critical role in cancer is miRNA-29, which has been shown to regulate the oncogenic process. miRNA-29 has been found to be a tumor suppressor, although there are some conflicting reports regarding its oncogenic effect [18]. On the other hand, in previous studies, miRNA-21 was found to have oncogenic effects and was upregulated in laryngeal carcinoma tissues [19]. Previous studies have shown that miRNA-9, as a tumor suppressor, is downregulated in patients diagnosed with oral squamous cell carcinoma (OSCC) [20].

In this study, the effect of miRNAs on the progression of laryngeal cancer was investigated by comparing FFPE tissues from patients with advanced and early stage laryngeal carcinoma. The expression levels of miRNA-21, miRNA802, miRNA29 and miRNA9, which are known oncogenic miRNAs, were compared in laryngeal carcinoma tissues.

## Materials and methods

This retrospective study was conducted with the approval of the Ethics Committee of the Faculty of Medicine of Tokat Gaziosmanpaşa University (16.03.2022/23-KAEK-063). Financial support was provided by the Coordination Unit for Scientific Research Projects of University of Tokat Gaziosmanpasa (2023/20) The study included 44 patients who underwent surgery (laryngectomy and/or direct laryngoscopy + biopsy) with a diagnosis of LSCC in our hospital between 2014 and 2024, and pathology specimens were available. Informed consent was not required for this type of retrospective study.

### Total RNA extraction

Total RNA extraction from paraffin tissue samples was performed using DiaRex^®^ Total RNA Extraction kit (Cat No: TR-0877, Diagen, Turkey). Briefly, 600 µL of Lysis (LBD) solution was added to the tissue scraped from the slide. The extraction process was performed according to the kit manufacturer’s instructions, and 30–50 µl of Total RNA was obtained at the end. Total RNAs were stored at -80 °C until the study was conducted.

## cDNA synthesis

The cDNA process was conducted using the extracted RNA samples, after calculating their amounts, via the SOLIScript^®^ RT cDNA synthesis KIT (SolisBIODYNE, Estonia) following the manufacturer’s instructions. Briefly; 2 µl 10x RT Reaction Buffer, 1 µl reverse transcription enzyme, 1 µl Oligo (dT) primer (or stem-loop primer, 0.5 µl dNTP MIX, 0.1 µl RNase inhibitor and variable amounts of distilled water were added together as a reaction mix so that the total volume was 20 µl. The PCR protocol included reverse transcription at 50 °C for 5 min and finally enzyme inactivation at 85 °C for 5 min.

## Real-time PCR

SolisFAST^®^ SolisGreen^®^ qPCR Mix (no ROX), 5X (SolisBIODYNE, Estonia) was used to determine gene expression levels.Thehousekeeping gene RNU6A (U6) was used as a normalizer in the study (Table 1). For the 1X PCR reaction, the total volume was 20 µl and consisted of 4 µl master mix, 5 µl mixB (containing 0.3mM forward and reverse primers), 6 µl dH2O, and 5 µl cDNA. The reaction was applied in a real-time PCR device (BioRAD CFX-96, Germany) with initial denaturation at 95 ^o^C for 5 min, 45 repetitions at 95 ^o^C for 5 s, 55 ^o^C (mir-9a/mir-21a/mir-29a/mir-802a) at 57 ^o^C (U6) for 30 s. Relative mRNA expression levels obtained for specific genes via the device were determined using the 2-ΔΔCt method with the R package (qpcrtools 1.0.1, ggpubr 0.6.0, dplyr 1.1.4, tidyverse 2.0.0, car 3.1-2). Outliers were identified and removed using the ROUT method in GraphPad Prism (Q = 1%).

**Table 1:** Oligonucleotides

Oligonucleotide Name	Oligonucleotide Sequence	Tm
mir-9a-F	CACGCAATAAAGCTAGATAACCG	55 C
mir-9a-Stem-loop	GTCGTATCCAGTGCAGGGCCGAGGTATTCGCACTGGATACGACACTTTC	
mir-21a-F	CACGCATAGCTTATCAGACTG	55 C
mir-21a-Stem-loop	GTCGTATCCAGTGCAGGGCCGAGGTATTCGCACTGGATACGACTCAACT	
mir-29a-F	CACGCATAGCACCATCTG	55 C
mir-29a-Stem-loop	GTCGTATCCAGTGCAGGGCCGAGGTATTCGCACTGGATACGACTAACGA	
mir-802a-F	CACGCACAGTAACAAAGATTC	55 C
mir-802a-Stem-loop	GTCGTATCCAGTGCAGGGCCGAGGTATTCGCACTGGATACGACACAAGG	
Universal-R	CCAGTGCAGGGCCGAGGTA	
U6_F	GCAAATTCGTGAAGCGTTC	57 C
U6_R	AGAAGCCATTAGTGTCCAAG	
U6-Stem-loop	GCTACCAGAAGCCATTAGTGTCCAAGTACTGCAATGAGAAGTTCGTCCTGCCGGGTAGCTTTTTTTTTTAAAAATAT	

**Table 2:** Descriptive statistics of hsa-miRNA expressions levels

hsa-miR-9a Expression Levels Parameter	Early-stage (*n* = 14)	Advanced-stage (*n* = 20)	*p*
Median	0.232	1.206	**0.0088**
IQR	0.006–1.188	0.429–12.84	
Mean ± SD	0.655 ± 0.937	7.264 ± 11.63	
Range (Min. - Max.)	0.001–3.046	0.005–40.98	
**hsa-miR-21a Expression Levels Parameter**	**Early-stage** (*n* = 15)	**Advanced-stage** (*n* = 19)	
Median	0.422	0.236	0.1931
IQR	0.006–3.826	0.070–11.84	
Mean ± SD	1.680 ± 2.261	8.413 ± 13.36	
Range (Min. - Max.)	0.001–7.340	0.001–38.74	
**hsa-miR-29a Expression Levels Parameter**	**Early-stage** (*n* = 18)	**Advanced-stage** (*n* = 18)	
Median	2.304	32.31	0.064
IQR	0.001–40.56	5.560–110.7	
Mean ± SD	24.30 ± 33.62	85.21 ± 136.8	
Range (Min. - Max.)	0.000-107.4	0.000-547.6	
**hsa-miR-802a Expression Levels Parameter**	**Early-stage** (*n* = 16)	**Advanced-stage** (*n* = 19)	
Median	1.007	9.287	**0.005**
IQR	0.033–6.001	1.018–46.37	
Mean ± SD	2.521 ± 3.194	26.49 ± 37.64	
Range (Min. - Max.)	0.001–8.847	0.055–117.4	

^*^ Mann Whitney U test was applied

## Results

**Fig. 1:**
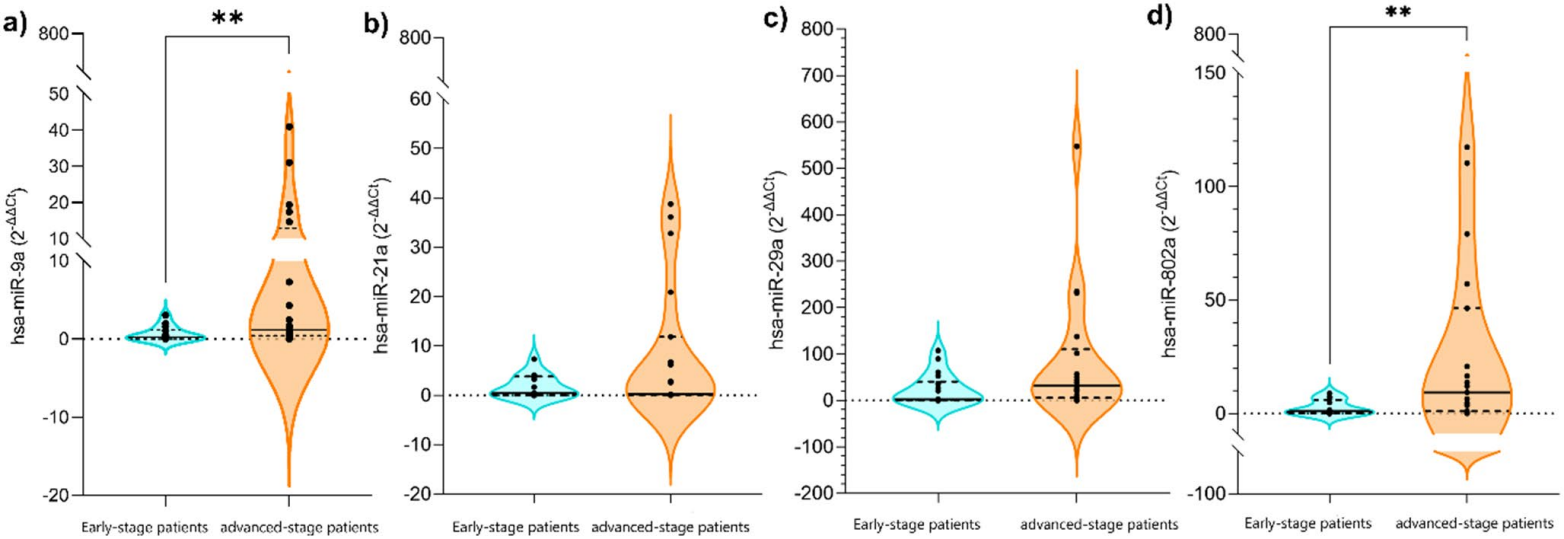
Statistical graph of miRNA expression levels in LCSS samples Graphs marked with ** are miRNA samples with statistically significant results

Expression of cDNA samples obtained from patient tissues was determined by real-time PCR. The expression level of hsa-miR-9a was significantly higher in the advanced stage patient group (Mdn = 1.206, IQR = 0.429–12.84) compared to the earlystage patient group (Table 2; Fig. 1a). The median difference between the groups was 0.974, with the advanced-stage patient group showing approximately 5.2-fold higher expression than the early-stage patient group. Initial sample sizes were *n* = 20 for the early stage patient group and *n* = 23 for the advanced stage patient group, with final analysis performed on *n* = 14 samples for the early stage patient group and *n* = 20 samples for the advancedstage patient group samples after outlier removal.

The expression level of hsa-miR-21a showed no significant difference between the advanced stage patient group (Mdn = 0.236, IQR = 0.070–11.84) and the early stage patient group (Table 2; Fig. 1b).The initial sample sizes were *n* = 20 for the early stage patient group and *n* = 23 for the treatment groups, with the final analysis performed on *n* = 15 samples for the early stage patient group and *n* = 19 samples for the advanced stage patient group samples after outlier removal.

The expression level of hsa-miR-29a showed no significant difference between the advanced stage patient group (Mdn = 32.31, IQR = 5.560–110.7) and the early stage patient group (Table 2; Fig. 1c). The initial sample sizes were *n* = 20 for the earlystage patient group and *n* = 23 for the advanced-stage patient group, with final analysis performed on *n* = 18 samples for the early stage patient group and *n* = 18 samples for the advanced stage patient group samples after outlier removal.

The expression level of hsa-miR-802a was significantly higher in the advanced stage patient group (Table 2; Fig. 1d) compared to the early-stage patient group (Mdn = 1.007, IQR = 0.033–6.001; Mann-Whitney U = 69.50, *p* = 0.005). The median difference between the groups was 8.280, with the advanced stage group having approximately 9.2-fold higher expression than the early stage group. Initial sample sizes were *n* = 20 for the early stage patient group and *n* = 23 for the advanced stage patient group, with final analysis performed on *n* = 16 samples for the early-stage patient group and *n* = 19 samples for the advanced stage patient group samples after outlier removal.

## Discussion

In our study, we investigated the relationship between miRNA expression and disease stage in cancer tissues from 20 early stage and 24 advanced stage LSCC patients. This is the first study to investigate tissue miRNA in relation to the progression of laryngeal cancer in the Turkish population. In this study, we demonstrated the effect of expression of miRNA-21, miRNA-802, miRNA-29 and miRNA-9, which are miRNAs with important effects on cancer pathogenesis, on progression in 44 patients with LSCC.

miRNAs have been detected in approximately 50% of mutated regions in tumor tissues, indicating that miRNAs are effective in tumorigenesis [21]. While many studies have compared the differences in miRNA expression between normal and cancerous tissues, primary lesions and metastatic/recurrent lesions, our study investigated the differences in miRNA expression between early stage and advanced stage patients [22]. Recent studies have identified many miRNAs that are upregulated (oncomirs) or downregulated (tumor suppressor miRs) in cancer tissues, and miRNA21 was found to be upregulated [23].

miRNA-21, which plays a role in cell differentiation, proliferation and apoptosis with its oncogenic effect, has been shown in many studies to cause tumor development. In patients diagnosed with LCSS, miRNA-21 can be used for early diagnosis, and some other studies have shown that it may be a biomarker for poor progression [24, 25]. It is believed that miR-21 may be associated with tumor progression in laryngeal cancer tissues with high expression levels. Studies have shown that cell cycle progression can be stopped when miR-21, an oncogenic microRNA overexpressed in laryngeal carcinoma tissues, is suppressed (26–27). In this study, which we conducted with the hypothesis that oncogenic microRNA-21 overexpressed in laryngeal carcinoma tissues may be a biomarker for poor prognosis, no significant difference was found between early stage and advanced stage patients.

Since miR-9 expression levels have been previously observed in multiple cancer types including colon cancer, nasopharyngeal cancer, melanoma and breast carcinoma, miR-9 is thought to be a tumor suppressor. When we look at the literature, miRNA9 levels in LCSS tissues were examined for the first time. Although many studies in recent years have shown that miR-9 has an oncogenic role, studies mostly show its tumor suppressor effect and that it is an important factor in the pathogenicity of various cancers, including head and neck cancers [28–30]. In our study, miR-9a expression level was approximately 5.2 times higher in the advanced stage patient group than in the early stage patient group, showing for the first time in the literature that it is an effective factor in the pathogenesis of LSCC patients.

Previous studies have shown that the expression level of miR-802 decreases in cancer tissues of tongue squamous cell carcinoma (TSCC), esophageal squamous cell carcinoma (ESCC), laryngeal squamous cell carcinoma (LSCC), gastric cancer (GC), colorectal cancer (CRC), pancreatic cancer (PC) and melanoma patients [31]. In our study, the expression level of miR-802a was approximately 9.2 times higher in the advanced-stage patient group than in the early-stage patient group, indicating for the first time in the literature that it is an effective factor in the pathogenesis of LSCC patients(p:0.005). Our study provides a novel finding for mi-802, indicating that its expression levels are increased in the advanced stage patient group.

In studies showing the effect of miR-29 on prognosis in cancer research, a parallel relationship was found between high miR-29 levels ​​and survival [32]. However, in our study, there was no significant difference was detected between the two groups(p: 0.064).

Abnormally expressed miRNAs target a wide range of genes and are strongly associated with cancer development, resistance to chemo-/radiotherapy and metastatic potential. The new and robust role of miRNAs in prognosis and therapeutic approaches is promising. miRNA expression levels may also provide valuable insight into resistance to CRT, thus improving the clinician decision making regarding cordectomy (partial laryngectomies)/radiotherapy in early stage laryngeal cancer and from complications associated with ineffective treatment modalities.

There are studies suggesting that miRNAs may be a guide for diagnosis and treatment in patients with LCSS [2, 4]. These biomarkers can be evaluated as targetedtreatment options in patients. In our study, the small number of patients and the inability to perform expression analysis from patients’ normal tissues are the most important main limitations of the study. However, the widespread use of organ-sparing protocols in advanced cancers has limited the number of patients by restricting our sample selection.

## Conclusıon

Our study revealed the expression levels of oncogenic miRNAs in laryngeal carcinoma tissues. We believe that our findings will lead to prospective studies that will reveal the relationship of expression levels with existing treatment modalities and their role in cancer progression.
